# Improving Cerebral Blood Flow after Arterial Recanalization: A Novel Therapeutic Strategy in Stroke

**DOI:** 10.3390/ijms18122669

**Published:** 2017-12-09

**Authors:** Mohamad El Amki, Susanne Wegener

**Affiliations:** Department of Neurology, University Hospital Zurich and University of Zurich, 8091 Zürich, Switzerland; susanne.wegener@usz.ch

**Keywords:** stroke, reperfusion, collaterals, hemorrhagic transformation, no-reflow and reocclusion

## Abstract

Ischemic stroke is caused by a disruption in blood supply to a region of the brain. It induces dysfunction of brain cells and networks, resulting in sudden neurological deficits. The cause of stroke is vascular, but the consequences are neurological. Decades of research have focused on finding new strategies to reduce the neural damage after cerebral ischemia. However, despite the incredibly huge investment, all strategies targeting neuroprotection have failed to demonstrate clinical efficacy. Today, treatment for stroke consists of dealing with the cause, attempting to remove the occluding blood clot and recanalize the vessel. However, clinical evidence suggests that the beneficial effect of post-stroke recanalization may be hampered by the occurrence of microvascular reperfusion failure. In short: recanalization is not synonymous with reperfusion. Today, clinicians are confronted with several challenges in acute stroke therapy, even after successful recanalization: (1) induce reperfusion, (2) avoid hemorrhagic transformation (HT), and (3) avoid early or late vascular reocclusion. All these parameters impact the restoration of cerebral blood flow after stroke. Recent advances in understanding the molecular consequences of recanalization and reperfusion may lead to innovative therapeutic strategies for improving reperfusion after stroke. In this review, we will highlight the importance of restoring normal cerebral blood flow after stroke and outline molecular mechanisms involved in blood flow regulation.

## 1. Introduction

According to the World Health Organization (WHO), each year, 15 million people suffer a stroke worldwide, of whom five million die and another five million show chronic disability [1,2]. Based on clinical evidence of better outcomes and reduced mortality, early revascularization is a critical process to rescuing salvageable tissue [3,4,5,6]. For the last 22 years, recanalization therapy has been induced by intravenous (i.v.) administration of recombinant tissue plasminogen activator (rt-PA) [3,7]; but recently, mechanical endovascular clot retrieval has also been approved, having shown effectiveness in several clinical trials [4,8]. Endovascular thrombectomy has revolutionized the management of stroke. It is one of the most effective treatments in medicine [4]. Indeed, although thrombolysis with rt-PA was the only effective treatment for ischemic stroke for a long time, recanalization rates of i.v. rt-PA have remained low in large artery occlusions [5,9,10,11,12]. For instance, in proximal middle cerebral artery (MCA), internal carotid artery (ICA) or basilar artery occlusions, recanalization with i.v. rt-PA was achieved in less than 20% of cases [11,13,14]. Today, by using thrombectomy approaches in large-vessel occlusion, substantial reperfusion is achieved in 70–80% of cases.

The recanalization rates of first-generation thrombectomy devices was quite similar to those with rt-PA. For instance, the first study of the thrombectomy devices Mechanical Embolus Removal in Cerebral Ischemia (MERCI), which was published in 2004, showed recanalization in only 43% of patients [15]; and the follow up MULTI MERCI trial showed a recanalization rate of 55% [16,17]. The Penumbra aspiration system was then developed as a second-generation device, and the results of the Penumbra Pivotal Stroke Trial were reported in 2008 [8]. This trial reported higher efficacy in opening occluded blood vessels compared to those reported for the MERCI device, and with equivalent safety (recanalization rates of 82%) [18]. In 2012, third-generation devices (SOLITAIRE and Trevo) showed very promising recanalization rates (92–94%) and clinical outcomes, as reported in the SOLITAIRE With the Intention For Thrombectomy trial (SWIFT), and Thrombectomy REvascularisation of large-Vessel Occlusions (TREVO 2) trial [19,20]. Today, with advanced thrombectomy technology, recanalization rates have dramatically improved, which is reflected in a better overall outcome for treated patients compared to early studies.

Despite this success, 30% to 68% of stroke patients still have an unfavorable clinical outcome, even after recanalization [21]. Similarly, after successful rt-PA thrombolysis, more than 50% of stroke patients do not show any sign of clinical improvement [5]. This “futile recanalization” that occurs after removal of the causative clot could be related to the occurrence of several vascular obstacles that stem from the vascular compartment of the brain, and which may hamper recovery of cerebral perfusion. For instance, clinical evidence suggests that some stroke patients do not show reperfusion even when recanalization is successful [22,23]. This has been termed “futile recanalization”, and has been attributed to the occurrence of the “no-reflow phenomenon” and/or arterial reocclusion [24,25]. The no-reflow phenomenon relates to the inability to reperfuse regions of the brain after ischemia, despite removal of the artery occlusion. The mechanism involves microvascular obstruction [26].

**Figure 1:**
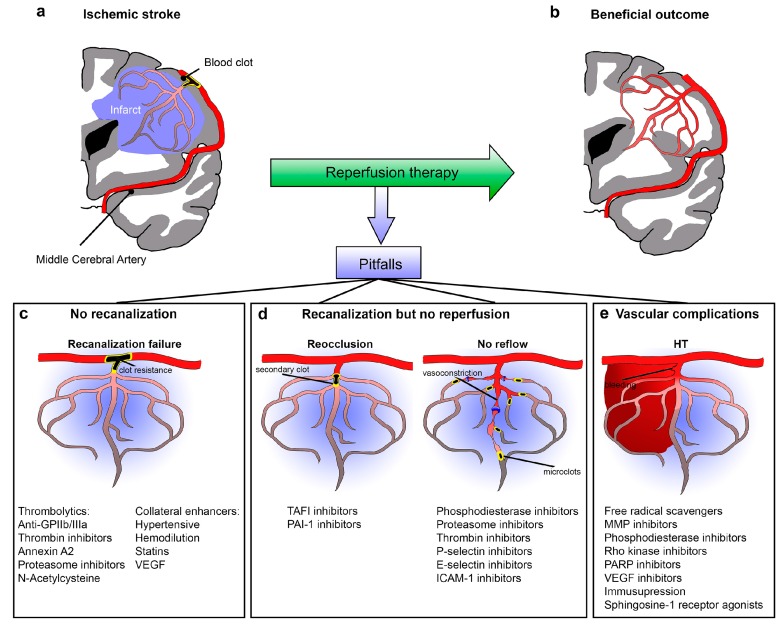
Vascular challenges for reperfusion therapy. (**a**) Schematic diagram of a coronal section of the brain. The middle cerebral artery (MCA) is occluded with a blood clot. The blue areas correspond to the infarct that could be saved with reperfusion therapy (**b**). The different possible pitfalls of reperfusion are shown as no recanalization (**c**), recanalization but no reperfusion and arterial reocclusion and no-reflow (**d**), and vascular complications: hemorrhagic transformation (**e**). In **c**–**e**, a summary of potential molecules involved in blood flow regulation is given for each scenario. Abbreviations: GPIIb/IIIa, glycoprotein IIb/IIIa receptors; VEGF, Vascular Endothelial Growth Factor; TAFI, Thrombin Activatable Fibrinolysis Inhibitor; PAI-1, Plasminogen Activator Inhibitor-1; ICAM-1, Intercellular Adhesion Molecule 1; MMP, Matrix Metalloproteinases; PARP, Poly-ADP-Ribose Polymerase.

In summary, clinicians are confronted with several obstacles when attempting recanalization therapy for stroke patients: (1) recanalization fails, (2) absence of reperfusion “no-reflow” or “reocclusion”, and (3) vascular complications such as hemorrhagic transformation (HT). These problems have been understudied, but with increasing use of thrombectomy in stroke, the need to understand the vascular and cerebral blood flow (CBF) changes associated with recanalization cannot be overemphasized. Each of these problems in reinstalling normal perfusion after stroke can also be approached from the molecular level, since several genes and proteins are induced after stroke and/or recanalization (Figure 1).

## 2. Recanalization Failure

### 2.1. Recanalization Rate

The first critical step in obtaining a favorable effect of reperfusion is to successfully re-open the occluded vessel and allow restoration of antegrade perfusion to the ischemic territory. Clinical data show that reperfusion therapies can result in different patterns of recanalization that can be complete, partial or absent (Table 1). To evaluate the degree of reperfusion, some mechanical thrombectomy studies have used the Thrombolysis In Cerebral Infarction (TICI) scoring system, which subdivides partial reperfusions into two different categories: 2a and 2b. The 2a partial grade means that less than two-thirds of the entire vascular territory is reperfused, while 2b recanalizations are almost complete, but slower than normal (Table 1). However, in some other clinical trials, recanalization has been evaluated according to the Thrombolysis in Myocardial Ischemia (TIMI) grading scale or the Thrombolysis in Brain Ischemia (TIBI), but using these scores does not provide any details about different partial patterns of recanalization.

After rt-PA thrombolysis, only 22% to 30% of patients have a complete recanalization; 23% to 48% have partial recanalization; and in 22% to 41% of patients, recanalization completely fails [14,27,28,29]. The use of mechanical thrombectomy in different clinical studies is associated with higher reperfusion success and less recanalization failure than rt-PA. For example, recanalization success reached more than 90% in the Extending the time for Thrombolysis in Emergency Neurological Deficits–Intra-Arterial (EXTEND-IA) randomized trial, and the rate of no recanalization was only 3% [30].

However, endovascular clot-retrieval therapy is only suitable for large arterial occlusions (internal carotid artery (ICA) and proximal M1 middle cerebral artery (MCA)), representing nearly 50% of stroke cases [33,34]. Smaller arteries present a real technical challenge for thrombectomy and recanalization, with intravenous thrombolysis remaining the most suitable strategy for these occlusions.

In addition to the approach used, recanalization success varies among stroke patients depending on the location and composition of the occluding clot as well as the collateral flow. Evidence from stroke patients shows that 20–30% of thrombi are resistant to endovascular retrieval [35]. Rt-PA also faces some highly resistant clots, depending on the location and clot composition. Indeed, large MCA clots are more resistant to thrombolysis, which leads to partial clot dissolution and greater tendency for arterial reocclusion [14]. The effect of rt-PA is dependent on contact with the surface of the clot; therefore, according to Riedel et al., short clots (length < 5 mm) are highly likely to be dissolved completely, but recanalization could fail in more than 99% of cases if the thrombotic clot length exceeds 8 mm [36]. Furthermore, various other factors, such as the experience of the interventionalist (number of cases performed) and the hospital setting, likely influence the success rate of recanalization.

### 2.2. Therapeutic Strategies for a Better “Clot-Buster”

Due to the resistance of some blood clots to rt-PA and endovascular thrombectomy, several thrombolytics such as tenecteplase, desmoteplase and reteplase have been developed and tested in clinical trials, but none of them were superior to rt-PA [37]. New therapeutic strategies are focusing on increasing the rates of recanalization by combining rt-PA with other agents, such as antiplatelets or direct thrombin inhibitors. Several antiplatelet antagonists of glycoprotein IIb/IIIa receptors (GPIIb/IIIa) have been evaluated as potential targets in either myocardial or cerebral ischemia for combination with thrombolysis [38]. Indeed, the administration of Tirofibran with rt-PA within 3 h of stroke results in a better recanalization rate in the MCA, and better clinical outcome in stroke patients [39]. The safety of the combination of Eptifibatide, another selective GPIIb/IIIa antagonist, with rt-PA was also evaluated in the CLEAR (Combined Approach to Lysis Utilizing Eptifibatide and rt-PA in acute ischemic stroke) trial showing that this combination of treatment can be safely performed in stroke patients [40]. Clinical data from 65 stroke patients show that the co-administration of rt-PA with Argatroban, a direct thrombin inhibitor, increases the fibrinolytic effect of rt-PA [41] and enhances recanalization rates, as shown in the TARTS (rt-PA Argatroban Stroke Study) clinical study [42]. In humans, although clinical trials evaluating the combination of antiplatelet therapy to thrombolysis were stopped early because of an increased rate of intracerebral hemorrhage [43,44], a recent retrospective analysis of stroke patients who received bridging thrombolysis with aspirin during endovascular intervention showed that the combination therapy does not increase the risk of bleeding complications [45].

Recombinant annexin A2 increases the catalytic efficiency of rt-PA in converting plasminogen to plasmin and enhances the thrombolysis efficacy of rt-PA, with improvement of neurological outcome in a rat model of stroke [46,47]. Combination treatment with a selective proteasome inhibitor, bortezomib, could increase the fibrinolytic activity of rt-PA, with an associated reduction in infarct volume and less HT compared to rt-PA alone [48,49]. Combination treatment of rt-PA with *N*-Acetylcysteine, a mucolytic drug with effects on cleavage of the von Willebrand Factor (VWF), exerts increased thrombolytic effects in a mouse model of stroke [50]. The influence of combined thrombolytics to rt-PA deserves further experimental and clinical investigations because, as discussed before, a high rate of patients still show no recanalization after treatment. Several antithrombotic treatments are already available in the clinic with a favorable safety profile in stroke, myocardial infarction and acute limb ischemia. Indeed, tirofiban has been proven to be safe in patients with ischemic stroke regarding the risk of hemorrhagic transformation in phase II-b studies. *N*-acetylcysteine is also safe, and is potentially a new, effective, thrombolytic treatment. Overall, the combined treatments with rt-PA are promising for thrombolysis of acute stroke, and clinical trials are now needed to evaluate their efficacy.

### 2.3. Collaterals

The collateral circulation is a physiologic pathway of endogenous vessels that maintain residual blood flow to brain regions distal to an arterial occlusion. Different sources of cerebral collateral flow exist, depending on the vessel size and location. The circle of Willis constitutes the main collateral network in the brain and is immediately available to maintain perfusion when a large artery is occluded. However, when occlusion occurs in an intracranial distal artery, the circle of Willis is unable to compensate the CBF reduction, and secondary collateral flow through leptomeningeal anastomoses is the principal alternative pathway [51]. Leptomeningeal anatomoses are cortical pial arteries that connect the major branches of the cerebral arteries—the anterior cerebral artery (ACA), the middle cerebral artery (MCA) and the posterior cerebral artery (ACA). The characteristic profile of leptomeningeal collaterals is that, in these vessels, blood can flow in both directions, allowing retrograde perfusion of adjacent territories and maintaining a viable region of brain tissue called the “ischemic penumbra”. In the ischemic penumbra, blood flow is sufficiently reduced to arrest physiological function, but not so completely as to cause irreversible cellular death [51]. Angiographic data grading collateral circulation in patients with stroke revealed that final infarct size [52,53] and functional outcome deficit vary with the presence or absence of a collateral network [54,55].

How can collateral flow impact the recanalization success?

In addition to its impact on infarct size and outcome, clinical data revealed that collaterals also influence the success of recanalization therapy in stroke patients [56,57,58]. Clinical studies show that collateral flow predicts the risk of HT after endovascular and thrombolysis therapies [59,60]. Patients with good collateral circulation show less risk of hemorrhagic complications after rt-PA thrombolysis or mechanical revascularization, and HT occurs more frequently in patients with poor collaterals (88.9% vs. 38.1%) [60,61]. Furthermore, the status of collateral flow is strongly related to the recanalization rate and reperfusion after revascularization [56,59]. For example, using the MERCI clot retriever, complete revascularization occurred in 14% of the patients with poor collaterals, in 25% of patients with good collaterals, and in 42% of patients with excellent collaterals [56]. After intravenous thrombolysis with rt-PA, patients with good collaterals showed higher rates of recanalization, in comparison to those with poor collaterals (61.8% vs. 28.1%) [61].

**Figure 2:**
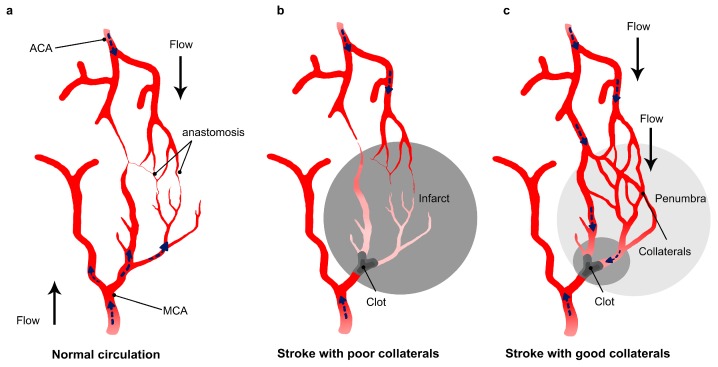
Impact of collateral flow on clot lysis and reperfusion. (**a**) Schematic drawing of the collateral network showing anastomoses between the middle cerebral artery (MCA) and anterior cerebral artery (ACA); (**b**) in stroke with a poor collateral network, the collaterals fail to fill and insufficiently compensate the flow reduction after arterial occlusion; (**c**) a collateral enhancement occurring in patients showing good collateral network. The flow in the collaterals changes direction and allows the thrombolytic to reach the drug from different sides.

There are several explanations for the beneficial effect of collaterals on recanalization rate in stroke patients. First, augmented collaterals may increase the delivery of thrombolytic agents to the clot (Figure 2). Thanks to robust collaterals, thrombolytics are able to reach the clot from different sides, which increases the efficacy of treatment, and therefore the rate of clot lysis. Furthermore, when the occlusive clot is dissolved, clinical data suggest that small fragments could migrate and dislodge into distal small arterial branches downstream of the primary occlusive lesion. Collateral flow could enhance the drug delivery to these distal branches and induce the dissolution of the fragmented proximal microclots. Additionally, in addition to the enhanced delivery of drugs to the clot site, collateral flow prevents impairment of vascular function and therefore improves reperfusion after recanalization therapy. Indeed, during cerebral ischemia, the damage is not restricted to neurons. Endothelial cells are affected as well [62,63,64]. Vascular damage occurring after stroke may lead to a worse result after recanalization in patients, as it facilitates edema formation and hemorrhagic transformation (HT). Therefore, collateral supply to the occluded vessel is crucial to reduce stroke induced damage and increase the chance of good reperfusion after recanalization.

### 2.4. Strategies to Enhance Collateral Circulation

Despite the important contribution of collateral circulation maintaining the penumbra and improving blood flow in ischemic brain tissue, the collateral network has been neglected in previous stroke studies. Recent studies have suggested that collateral flow could be enhanced by adjusting head position and intravenous fluid support, while others have tested pharmacological-induced hypertension, vasodilation and hemodilution [65,66].

The beneficial effects of induced hypertension have been confirmed in animal models of stroke [67]. Intravenous infusion of phenylephrine increased blood pressure, and was associated with reduced infarct volume, as well as improved reperfusion in rat and rabbit animal models of stroke [68,69]. However, although mild hypertension induced during acute stroke appears to be protective, chronic hypertension paradoxically worsens stroke outcome [70]. The safety and efficacy of induced hypertension using phenylephrine in patients with ischemic stroke is under investigation in the clinical trial SETIN-HYPERTENSION (The Safety and Efficacy of Therapeutic Induced HYPERTENSION in acute ischemic stroke) [71]. As a hemodiluting agent, albumin has been shown to increase collateral formation and enhance reperfusion after distal MCA occlusion in mice [72]. However, the clinical trial ALIAS (Albumin in Acute Ischemic) showed that treatment with intravenous albumin in stroke patients was not associated with improved outcome at 90 days, and was associated with increased rates of HT and pulmonary edema [73]. Collateral flow improvement by chemokines and growth factors, including vascular endothelial growth factor (VEGF) and statins, has also been evaluated in ischemic stroke. Harrigan et al. reported that VEGF infusion in middle cerebral artery occlusion (MCAO) rats increased the vascular density in a dose-dependent manner and minimized the associated brain edema after ischemic stroke [74]. Ovbiagele et al. have shown that statins may enhance the collateral supply in stroke and patients using statins as pretreatment have significantly higher collateral scores than the non-statin users [75]. Statins are safe in stroke, as has been shown in clinical trials, and the combination of rt-PA to simvastatin in patients is associated with low rates of bleeding complication (STAR Stroke Treatment With Acute Reperfusion and Simvastatin trial) [76]. However, the STARS trial was underpowered for detecting differences in simvastatin efficacy because of the low recruitment rates. To the best of our knowledge, there is still no clinical data supporting the use of medical therapies targeted at the enhancement of the collateral network.

## 3. No Reperfusion Despite Recanalization Successes (Futile Recanalization)

As mentioned above, successful recanalization does not consistently lead to better outcomes in stroke patients, as more than 50% of patients with successful rt-PA thrombolysis or thrombectomy have an unfavorable outcome [5,21]. This “futile recanalization”, which occurs after removal of the causative clot, has been attributed to “arterial reocclusion” and to the “no-reflow phenomenon” [24,25].

### 3.1. Arterial Reocclusion

Arterial reocclusion is defined as a subsequent occlusion of a target vessel after initial recanalization. Clinically, the occurrence of the vascular reooclusion is characterized by a brief initial clinical improvement (due to successful recanalization) followed by a deterioration (due to reocclusion) in the absence of intracranial hemorrhage. In the National Institute of Neurological Disorders and Stroke (NINDS) trial, 13% of patients treated with rt-PA experienced an early clinical deterioration after an initial improvement, representing arterial reocclusion [77]. In other clinical studies, arterial reocclusion has been documented in about 20 to 34% of rt-PA-treated patients after successful thrombolysis [28,29]. This high proportion of arterial reocclusion (higher than the rate of HT) observed in most academic stroke centers emphasizes the need to better understand this vascular complication. Early reocclusion following successful recanalization is associated with a significantly poorer outcome at 3 months and a higher in-hospital mortality compared to patients without reocclusion [28]. However, patients with reocclusion still have better long-term outcomes and less mortality than patients without any early recanalization [28]. These data suggest that even a brief recanalization before arterial reocclusion induces a beneficial effect in stroke patients.

Subacute reocclusion occurs within the first 2 h after recanalization in cerebral vessels following the administration of thrombolytic agents or endovascular therapy [14,28,78]. Arterial reocclusions are more frequent when the recanalization is incomplete. Indeed, clinical data show that partial clot dissolution after thrombolysis is associated to a greater tendency for reocclusion [14]. Several factors are involved in the mechanisms of arterial reocclusion such as migration of dissolved clots that occlude distal arterial branches or reformation of new thrombus. The reformation of new thrombus at the site of occlusion have been studied in the coronary circulation as well as the cerebral circulation [79]. Although thrombolytic therapy is able to dissolve occlusive thrombi, it creates a procoagulant environment by generating plasmin [80]. The plasmin activates platelets and generates thrombin, increasing the likelihood of vessel reocclusion [80]. The use of endovascular clot retrieval may also activate the clotting cascade because of a disruption of atherosclerotic plaques or endothelial erosion that triggers platelet activation, adherence and aggregation, and also the exposure of tissue factor [79,80].

### 3.2. Therapeutic Targets against Arterial Reocclusion

Currently, there is limited information about detailed mechanistic aspects of the reocclusion process. As arterial reocclusion could be a major contributor to futile recanalization, more preclinical investigations are necessary to assess possible molecular pathways and therapeutic targets related to reocclusion scenarios. These strategies should focus on the activation of the coagulation cascade and the infiltration of procoagulation factors such as TAFI (thrombin-activatable fibrinolysis inhibitor) and PAI-1 (plasminogen activator inhibitor-1) and on the vascular dysfunction and constriction after reperfusion. All of these factors may be able to impact reocclusion rate after reperfusion, but more studies are necessary to arrive at a concrete recommendation.

### 3.3. No-Reflow

No-reflow describes a failure of microcirculatory reperfusion despite clot removal. Ames and coworkers were the first to describe the “no-reflow” phenomenon in 1968. They described an incomplete cerebral blood-flow restoration after mechanical recanalization in a rabbit model of cerebral ischemia [81]. Angiograms from stroke patients confirmed the existence of no-reflow in the clinic; in some cases, although clots were completely dissolved and the vascular patency restored, the reperfusion in stroke patients was non-existent [82,83]. Furthermore, data from stroke patients confirm that tissue reperfusion is a more accurate predictor of outcome after thrombolysis than recanalization [23]. When the post-stroke microvascular no-reflow occurs, it attenuates the beneficial impact of reperfusion, resulting in poor clinical outcomes [84,85]. Although clinical data show microvascular perfusion failure after recanalization, little is known about the mechanisms of no-reflow, because it is difficult to assess, both in clinical imaging and in experimental models [86]. Experimental data from a mouse model of stroke demonstrated that after successful intravenous thrombolysis, about half of the capillaries remain constricted [26]. Narrowing of the microvascular lumen was attributed to a compression caused by swollen astrocyte end feet and endothelial cells [87,88]. Several years later, Yemisci and colleagues showed that pericytes are also involved in the capillary constriction, leading to a reduced lumen and an incomplete microcirculatory reperfusion [26]. Constricted microvessels after stroke show narrowed lumina, entrapped erythrocytes, leukocytes and fibrin-platelets deposits. After recanalization, the fibrin and platelets deposit in the capillaries are associated to the areas with remaining hypoperfusion in rat brain after cerebral ischemia [89].

In addition to the microvascular constriction, a primary clot can break into fragments that migrate and occlude smaller arterial branches downstream of the primary occlusive lesion [90]. Microclots have been found in brain microvessels of stroke patients who died within a month after the stroke onset [91]. Another important factor contributing to the no-reflow phenomenon during reperfusion is the impairment of vascular patency after stroke. Cerebral ischemia is known to impair the dilation ability of arterioles in response to endothelium-dependent vasodilators, such as nitric oxide (NO) and acetylcholine (Ach) [62,92]. Reduced endothelial vasoreactivity was reported after cerebral ischemia/reperfusion, and could contribute to the impairment of blood flow restoration [62]. Permanent and transitory cerebral ischemia alter the ability of relaxation in the microvascular bed and the perfusion in the downstream capillary. This altered vascular reactivity is due to a reduced release of nitric oxide (NO) after stroke and reperfusion [93,94,95,96]. The reduced release of NO could lead to pericyte contraction and erythrocyte entrapment [26].

### 3.4. Therapeutic Strategies for Treatment of No-Reflow

At present, there are no specific therapies targeting no-reflow after stroke. In myocardial infarction, no-reflow is a field of intense research, and the treatment of no-reflow is based on vasodilators like adenosine and verapamil, GPIIb/IIIa receptor blockers, intra-coronary Ca^2+^ blockers, as well as clearance of microvascular plugging [97]. In cerebral ischemia, very few therapies have been tested, but it has been suggested that reducing microvascular clogging by inhibiting fibrin or platelets and leukocyte adherence or vascular inflammation restores microcirculation, reduces no-reflow, and improves stroke outcome in animal models [98,99,100,101]. However, these strategies have never been evaluated in clinic, due to the difficulties in assessing the microvascular reperfusion in stroke patients. Cilostazol, a phosphodiesterase inhibitor acting as an antiplatelet agent, reduced the no-reflow and HT induced by rt-PA, via maintenance of microvascular integrity in a MCAO mouse model [102]. Administration of a direct thrombin inhibitor, argatroban, enhances the recanalization rates induced by rt-PA by preventing the no-reflow [41]. Administration of Pioglitazone, an activator of peroxisome proliferator-activated receptor-gamma (PPARγ), reduces the no-reflow phenomenon in microvessels after MCAO in rats [103]. Furthermore, adhesion molecule-blocking antibodies that inhibit leukocyte adhesion, such as P-selectin, E-selectin, and ICAM-1, also improve the rt-PA induced reperfusion in post-ischemic cerebral mouse brains by preventing no-reflow [98,100,101]. Due to the multiple functions of pericytes in the microcirculatory system, development of drugs targeting pericytes is a promising new strategy for the prevention and treatment of the no-reflow phenomenon. Pericyte dilatation could be mediated mainly by NO [104]. Moreover, some signals of increased energy utilization, including lactates, adenosine and low pH, could also be investigated for their relaxing properties on pericytes [105].

## 4. Reperfusion with Vascular Complications

In addition to recanalization and reperfusion failure, tissue hemorrhage may occur after recanalization therapy in stroke patients. This is one of the most feared complications of stroke thrombolysis and thrombectomy, because it is potentially life threatening.

### 4.1. Hemorrhagic Transformation

Hemorrhagic transformation (HT) refers to bleeding into an ischemic area in a primarily ischemic stroke. Symptomatic HT occurs in a significant proportion of patients, and is associated with neurological deterioration and increased mortality. The HT rate after cerebral ischemia varies between 10% and 40%, depending on individual factors such as age, blood glucose level, and the time window allowed for the initiation of the therapy [106,107]. Thrombolysis with rt-PA increases the rate of HT by 6–10 fold [6,108]. However, the increase of HT with rt-PA is not always clinically relevant, and it is still a matter of debate as to how rt-PA could enhance the extent of HT, and at the same time improve patients’ functional outcomes [109,110]. Indeed, clinical data from both European Cooperative Acute Stroke Studies (ECASS) 1 and 2 indicate that, although rt-PA increases the risk of HT, it reduces the overall risk for disability and death by 6% and 8%, respectively [111,112]. To explain this contradiction, Von Kummer et al., suggested that the risk of HT after thromobolysis has been overestimated, and that some imaging-defined HT lesions represent reperfusion following successful and early recanalization after administration of rt-PA [110,113,114].

Reperfusion of a severely ischemic tissue may lead to deleterious consequences, known as the reperfusion injury, which leads to the disruption of the blood brain barrier (BBB). Reperfusion increases the production of oxygen radicals, which involves formation of hydrogen peroxide, hydroxy radicals, and superoxide [115,116,117]. These radicals result in increased BBB permeability, disruption of endothelial cell membranes, increased platelet aggregability and alterations in vascular response to CO_2_ [116]. Accordingly, it is now well established that reperfusion is a key factor in HT [118]. By using magnetic resonance imaging in stroke patients, it was shown that rt-PA is associated with BBB breakdown, which is correlated to HT [119,120].

Furthermore, beyond its role in thrombolysis and reperfusion injury, rt-PA may promote HT through other mechanisms, such as increasing metalloproteinase activity and the low-density lipoprotein receptor-related protein (LRP) receptor signaling [118,119,121]. rt-PA increases the matrix metalloproteinases MMP-2 [122], MMP-3 [123] MMP-9 levels in the brain [124]. Metalloproteinases (MMPs) are responsible for the degradation of the extracellular matrix and vascular basement membrane that leads to BBB breakdown. The activity of the MMPs increases after rt-PA administration, especially MMP-9, which has been shown to be elevated in venous blood from stroke patients that received rt-PA treatment [125,126]. Furthermore, rt-PA can promote the degranulation of neutrophils into the blood. Since neutrophils are the main source of MMP-9, the rt-PA induced degranulation increase the MMP level and the thrombolysis-related brain bleedings [127]. rt-PA is also capable of interacting with the LRP on endothelial cells and enhance the release of MMP-3 and MMP-9 as well as the detachment of astrocytic end-feet leading to a dysregulation of the BBB [128,129].

### 4.2. Strategies against Hemorrhagic Transformation

Several therapies have been evaluated for the prevention of HT induced by reperfusion therapies. The molecular targets that have been evaluated for HT prevention include inhibiting MMPs, reducing oxygen radicals, and modulating targets that affect BBB permeability.

Free-radical scavengers aiming to reduce stress oxidants, such as edaravone, uric acid and NXY-059 protect the BBB and reduce HT induced by rt-PA in animal models of cerebral ischemia [130,131,132,133]. However, targeting the stress oxydant failed to show beneficial effects in clinical trials, and treated stroke patients did not show any signs of clinical improvement [131,133]. Inhibition of MMP by pharmacological drugs such as Batimastat (BB-94) and minocycline reduces BBB permeability and the rate of rt-PA related in rats and rabbit models of stroke [134,135]. Furthermore, in experimental models of stroke, several therapies also showed reduction of the rt-PA-related hat, such as cilostazol [102], fasudil (rho kinase inhibitor) [136], fingolimod (sphingosine 1-phosphate receptor agonist) [137], polyADP ribose polymerase (PARP) inhibitors [138,139], FK506 (tacrolimus, immusupressive drug) [140], and VEGF inhibition [141,142]. Although preclinical studies have demonstrated the potential effect of several drugs to reduce the rt-PA induced HT, few are under investigation in clinic. Edaravone stroke trial PROTECT 4.5 has shown that the frequency of intracerebral HT is lower with combined rt-PA to edaravone than with rt-PA alone [143]. Albumin, minocycline and simvastatin are also under investigation in clinical trials [144].

## 5. Conclusions and Future Direction

After cerebral ischemia, blood flow disruption limits the delivery of glucose and oxygen to neurons, causing a cascade of energy failure events, and a complex series of biochemical events including neuroinflammation, excitotoxicity, oxidative and nitrative stress, Ca^2+^ influx and proapoptotic cascade activation [145,146]. Decades of research has focused on the neural consequences after stroke by searching for new neuroprotective strategies, but translation into clinical therapies has been difficult [147]. Researchers have outlined a variety of reasons for this clinical failure; mainly lack of efficacy, intolerable side effects of the treatments, or issues regarding quality and conduct of experimental research studies [148,149]. However, the vascular injury of stroke per se has been neglected, thus far. It is possible that neuroprotective therapies have failed in humans because the damaged vascular network is unable to deliver the necessary nutrients and treatment to the tissue at risk, thus also hampering neuroprotection.

There are theoretical reasons and evidence from animal experimentation, as well as clinical trials, suggesting that CBF restoration is a key determinant of better outcomes in stroke patients. Accordingly, when reperfusion therapy is executed, not only should the clot be removed, but the vessels should also be protected to restore physiological reperfusion. However, different states of vascular patency and function after recanalization in stroke contribute to treatment success: some could hamper the benefit of recanalization (no-reflow, reocclusion and HT) and others could enhance it (collaterals). Some vascular phenomena such as HT have been widely investigated in stroke, while others, such as the reocclusion, collaterals and no-reflow, remain relatively understudied. Therefore, there is an urgent need to gain additional mechanistic insight into the molecular events that are triggered by reperfusion, and which could be exploited therapeutically. Understanding the role of vascular pathology after stroke should be a prioritized research goal, in order to increase the chance of successful translation of treatments into the clinic and, most of all, to improve patients’ recovery.

## Figures and Tables

**Table 1:** Recanalization patterns after rt-PA thrombolysis in clinical studies.

Therapy		Recanalization			
		Successful			Failed
	References	Complete	Partial (2b)	Partial	No Recanalization
Thrombolysis	Christou et al., [27]	30%		40%	30%
	Alexandrov et al., [28]	30%		48%	22%
	Rubiera et al., [14]	22%		37%	41%
	Saqqur et al., [29]	27%		23%	37%
Endovascular Thrombectomy	MERCI [31]	24%		42%	33%
	Penumbra [18]	18%		54%	28%
	TREVO [20]	14%		78%	8%
	MR CLEAN [32]	24%	35%	27%	14%
	EXTEND-IA [30]	48%	38%	10%	3%

## Abbreviations

rt-PA	Recombinant tissue Plasminogen Activator
HT	Hemorrhagic Transformation
CBF	Cerebral Blood Flow
WHO	World Health Organization
LRP	Lipoprotein Receptor-related Protein
ICH	Intracerebral Hemorrhage
MCA	Middle Cerebral artery
ACA	Anterior Cerebral Artery
PCA	Posterior Cerebral Artery
ICA	Internal Carotid Artery
BBB	Blood Brain Barrier
MMP	Matrix Metalloproteinase
